# Structures of Reverse Transcriptase Pre- and Post-Excision Complexes Shed New Light on HIV-1 AZT Resistance

**DOI:** 10.3390/v3010020

**Published:** 2011-01-18

**Authors:** Walter A. Scott

**Affiliations:** Department of Biochemistry and Molecular Biology, University of Miami Miller School of Medicine, P.O. Box 016129, Miami, FL 33101, USA; E-Mail: wscott@med.miami.edu; Tel.: +1-305-243-6359; Fax: +1-305-243-3955

**Keywords:** reverse transcriptase, HIV, nucleotide excision, AZTppppA, AZT resistance

## Abstract

HIV-1 resistance to 3′-azido-2′,3′-deoxythymidine (AZT, zidovudine) results from mutations in reverse transcriptase that increase the ability of the enzyme to excise AZT-monophosphate after it has been incorporated. Crystal structures of complexes of wild type and mutant reverse transcriptase with double-stranded DNA with or without the excision product, AZT adenosine dinucleoside tetraphosphate (AZTppppA), have recently been reported [1]. The excision-enhancing mutations dramatically change the way the enzyme interacts with the excision product.

In a recent paper [1], investigators at Rutgers University in Piscataway, New Jersey, The National Cancer Institute in Frederick, Maryland, and The University of Missouri in Columbia, Missouri, described several crystal structures of HIV-1 reverse transcriptase (RT) containing resistance mutations to 3′-azido-2′,3′-deoxythymidine (AZT, zidovudine) that provide new molecular insights into a major mechanism of resistance of HIV-1 to AZT, the first antiretroviral drug approved by the U.S. Food and Drug Administration for use in the AIDS epidemic.

Several nucleoside analogs lacking the 3′ OH have proven to be useful therapeutic agents against HIV infection; however, prolonged use of these drugs leads to selection of resistance mutations. Resistance to AZT is not usually associated with reduced ability of the mutant RT to bind AZT triphosphate (AZTTP) or to incorporate AZT monophosphate (AZTMP). Instead, the mutant RTs incorporate AZTMP as efficiently as wild type RT, but have increased ability to excise the chain-terminating residue after it has been incorporated [2,3]. Excision is accomplished by transfer of the AZTMP moiety to an acceptor substrate, removing the block to elongation so that the DNA chain is free to be elongated with natural nucleotides. Enhanced excision by these mutants is not limited to AZTMP and can extend to other nucleoside RT inhibitors, but resistance is typically much greater for AZT than for other chain terminators [4]. The mutants are widely referred to as thymidine analog resistance mutations (TAMs).

Excision occurs through a mechanism related to pyrophosphorolysis, the reversal of the DNA polymerization reaction. In pyrophosphorolysis, the 3′-terminal nucleotide on the primer strand is transferred to pyrophosphate forming the corresponding nucleoside triphosphate. HIV-1 RT has a unique ability to use cellular nucleoside triphosphates in place of pyrophosphate in this reaction forming dinucleoside tetraphosphate as the excision product [5]. The nucleoside triphosphate most readily available for excision in the intracellular environment is ATP, and it is considered the most likely candidate for acceptor substrate in the *in vivo* reaction. The product of ATP-dependent excision of AZTMP is AZT adenosine dinucleoside tetraphosphate (AZTppppA) and the formation of AZTppppA is enhanced in *in vitro* reactions with mutant RTs containing TAMs [3]. The new crystal structures reported by Tu *et al.* [1] provide direct structural evidence to explain enhanced excision by the mutant RTs.

These authors have described ternary complex crystal structures consisting of wild type or AZT-resistant mutant RT, 27:21-mer double-stranded DNA, and AZTppppA, using a previously reported cross-linking strategy [6,7]. Cross-linking was introduced between a cysteine introduced by a Q258C mutation in the large subunit of RT (p66 subunit) and an N^2^-thioalkyl tether attached to an internal G in the primer strand. The investigators also introduced dideoxy-AMP at the 3′ terminus of the primer strand to prevent reincorporation of AZTMP. The primer-template contains a template dA in the polymerase active site allowing it to base-pair with an incoming T analog. AZTppppA was prepared synthetically and bound in the dNTP-binding site on the enzyme to form a stable closed ternary complex. The mutant RT contained five of the most prevalent TAMs (M41L, D67N, K70R, T215Y and K219Q). Structures were obtained for ternary complexes with wild type RT and mutant RT at resolutions of 3.15 Å and 3.2 Å, respectively. The investigators also determined structures for two binary complexes containing the mutant enzyme in complex with AZTMP-terminated dsDNA and a structure for the unliganded mutant RT.

The most remarkable finding from these structures is that the AMP portion of AZTppppA occupies very different positions in wild type and mutant RT (Figure 1). The AMP binding site is well defined in the mutant complex, but much more variable in the wild type complex. In both of these complexes AZTppppA is recognized as an analog of TTP and the AZTTP portion of the molecule superimposes closely with TTP in the ternary complex containing wild type RT, dsDNA and TTP [8]. The AMP portion of AZTppppA finds a place to bind near site I in wild type RT (Figure 1), but does not form distinctive interactions with RT residues. By contrast, the primary TAMs (T215Y and K70R), form a new binding site for AMP on the surface of the enzyme that causes the AMP moiety to bind more tightly at an alternative position (site II), about 10 Å from the binding site in the wild type enzyme. AZTppppA binding to both mutant and wild type enzyme is dominated by the interaction with the AZTTP portion of the molecule, which is not much affected by the TAMs. These results are consistent with previous reports that both wild type and mutant enzymes have little ability to distinguish between TTP and AZTTP, and that AZTppppA is a good substrate for AZTMP incorporation by the mutant enzyme (in fact it is a better substrate than AZTTP [9]), but a poor substrate for wild type RT.

The contributions of the primary TAMs to ATP-dependent excision agree with predictions made previously on the basis of molecular modeling [10,11]. The adenine ring of the AMP portion of AZTppppA interacts with tyrosine 215 through a π–π stacking interaction, and the ribose and α phosphate portions of AMP interact with arginine 70 through hydrogen bonding between the guanidinium of arginine, the 3′ and 5′ oxygen atoms in the ribose ring, and the α phosphate. These observations establish a new binding site for AMP in the mutant enzyme that does not exist in the wild type enzyme. Secondary TAMs are mutations that confer little resistance by themselves but increase the level of resistance when present together with primary TAMs. The secondary TAMs, D67N and K219Q, can be largely understood as enhancing the ability of the AMP structure to occupy site II. Glutamine-219 in the mutant enzyme interacts with the 4′ oxygen in the ribose ring of AMP when it occupies site II but, perhaps more importantly, wild type lysine at position 219 forms an ion pair with aspartate at position 67 [10] that could affect the access to site II. Mutation to a neutral residue at either position 219 or 67 would prevent the formation of the salt bridge and increase access to this site. Loss of interaction between residues 219 and 67 also removes a constraint on the position of the β3-β4 loop in the fingers portion of the enzyme (residue 67 is at the tip of that loop). Excision is known to be enhanced by other mutations that alter the β3-β4 loop including dipeptide insertions following residue 69 [12–15].

The contribution of the M41L mutation is still something of a mystery although it is arguably the most important secondary TAM based on the high frequency with which the M41L/T215Y combination is observed [16]. This residue is not close enough for direct interaction with any portion of the AZTppppA ligand in the mutant RT ternary complex structure; however, in infected cells, the M41L mutation substantially enhances AZT resistance when the T215Y mutation is also present. The leucine at position 41 is 3.9 Å from phenylalanine-116 and the authors speculate that this interaction may stabilize binding between the 3′-azido group at the primer terminus and the binding pocket formed by alanine-114, tyrosine-115, phenylalanine-116 and glutamine-151, which would favor binding of the primer terminus in the dNTP binding site (N-site). Preferential N-site occupancy by the mutant enzyme could help explain enhanced excision activity since the primer terminus must be located in the N-site for excision to occur.

Tu *et al.* [1] also report structures of binary complexes in which the primer-terminating AZTMP occupies the pre-translocation (N-site) or post-translocation (P-site) positions on the mutant RT. In the P-site complex, the p66 fingers subdomain residues occur in an “open” configuration as they do in other binary complex structures including the wild type N-site and P-site complexes previously published by these investigators [17]. By contrast, the fingers subdomain occurs in a “closed” configuration in the mutant N-site complex giving it an overall structure that is similar to the ternary complexes containing the excision product. The authors suggest that the closed structure may depend on the presence of a primer-terminal AZTMP in the N-site. AZTMP-terminated primer-templates have previously been shown to bind preferentially in the pre-translocation position on the wild type enzyme [18,19]. The authors point out that the availability of a closed N-site binary complex and a closed ternary complex containing AZTppppA provides a unique opportunity to model the transition state in the excision reaction. The resulting model suggests that the transition state intermediate matches the intermediate formed during DNA polymerization. The attacking γ phosphate of the ATP excision substrate is positioned ∼ 3 Å from the α phosphate of the primer terminal AZTMP and the attacking γ phosphate oxygen assumes an almost collinear position with respect to the scissile phosphate bond of AZTMP prior to the attack.

In summary, our previous understanding of the mechanism of AZT resistance was based largely on molecular modeling using the ternary complex containing WT RT, dsDNA and the incoming dNTP [8]. The new structures confirm many of the conclusions from that modeling; however, they have also provided some surprises. A new binding site for the AMP moiety is formed in the mutant enzyme that was not present in the wild type enzyme, and the pre-excision binary complex of mutant RT and AZTMP-terminated primer-template occurs in a “closed” configuration. Some questions remain: Notably, why does the selection of TAMs divide into two different pathways—the more common TAM-1 pathway (leading to M41L, L210W and T215Y) and the less common TAM-2 pathway (leading to D67N, K70R, T215F and K219Q/E) [20]? And why are the K70R and M41L mutations antagonistic to each other during the early stages of selection [21]? Nonetheless, the availability of these new structures provides an invaluable resource for the design of drugs that target the resistant enzyme. These results may lead to the development of drugs that increase the potency of a wide variety of nucleoside RT inhibitors by preventing their excision.

## Figures and Tables

**Figure 1:**
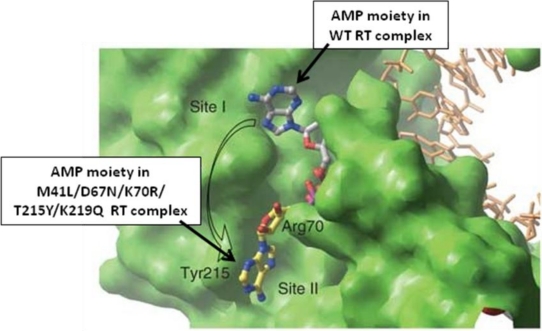
Comparison of binding positions of the AMP portion of AZTppppA in ternary complexes formed by wild type RT (Site I) and the AZT-resistant mutant RT (Site II). The molecular surface is shown for the AZT-resistant enzyme and the positions of the primary TAMs (arginine-70 and tyrosine-215). AMP carbons in the wild type complex are shown in grey and in the mutant enzyme complex are shown in yellow. The positions of the two AMPs are ∼ 10 Å apart. Reprinted with permission from [1].
